# Extracellular electrophysiology on clonal human β-cell spheroids

**DOI:** 10.3389/fendo.2024.1402880

**Published:** 2024-05-31

**Authors:** Emilie Puginier, Karen Leal-Fischer, Julien Gaitan, Marie Lallouet, Pier-Arnaldo Scotti, Matthieu Raoux, Jochen Lang

**Affiliations:** Univiversity of Bordeaux, CNRS, Bordeaux INP, Laboratoire de Chimie et Biologie des Membranes CBMN, UMR 5248, Pessac, Bordeaux, France

**Keywords:** spheroids, extracellular electrophysiology, microelectrode array, islets, insulin, EndoC-βH1, EndoC-βH5, INS-1 cells

## Abstract

**Background:**

Pancreatic islets are important in nutrient homeostasis and improved cellular models of clonal origin may very useful especially in view of relatively scarce primary material. Close 3D contact and coupling between β-cells are a hallmark of physiological function improving signal/noise ratios. Extracellular electrophysiology using micro-electrode arrays (MEA) is technically far more accessible than single cell patch clamp, enables dynamic monitoring of electrical activity in 3D organoids and recorded multicellular slow potentials (SP) provide unbiased insight in cell-cell coupling.

**Objective:**

We have therefore asked whether 3D spheroids enhance clonal β-cell function such as electrical activity and hormone secretion using human EndoC-βH1, EndoC-βH5 and rodent INS-1 832/13 cells.

**Methods:**

Spheroids were formed either by hanging drop or proprietary devices. Extracellular electrophysiology was conducted using multi-electrode arrays with appropriate signal extraction and hormone secretion measured by ELISA.

**Results:**

EndoC-βH1 spheroids exhibited increased signals in terms of SP frequency and especially amplitude as compared to monolayers and even single cell action potentials (AP) were quantifiable. Enhanced electrical signature in spheroids was accompanied by an increase in the glucose stimulated insulin secretion index. EndoC-βH5 monolayers and spheroids gave electrophysiological profiles similar to EndoC-βH1, except for a higher electrical activity at 3 mM glucose, and exhibited moreover a biphasic profile. Again, physiological concentrations of GLP-1 increased AP frequency. Spheroids also exhibited a higher secretion index. INS-1 cells did not form stable spheroids, but overexpression of connexin 36, required for cell-cell coupling, increased glucose responsiveness, dampened basal activity and consequently augmented the stimulation index

**Conclusion:**

In conclusion, spheroid formation enhances physiological function of the human clonal β-cell lines and these models may provide surrogates for primary islets in extracellular electrophysiology.

## 1 Introduction

Pancreatic islets are important in nutrient homeostasis and their dysfunction leads to a major metabolic disease, diabetes (1, 2). Glucose metabolism in β-cells leads to membrane depolarization and calcium influx that triggers insulin secretion (3). Studies on primary islet cells are hampered by the relative scarceness of native material, especially those of human origin, which moreover differ in several important aspects from rodent islet cells (3) and exhibit a high degree of variability (4). Consequently, clonal β-cell lines still provide very useful models and this approach has been considerably improved by the establishment of a human β-cell line, EndoC-βH1 cells and their derivatives (5–9).

A number of parameters such as ultrastructure, gene expression, survival and secretion has been investigated in the different EndoC-βH cell lines. However, electrical activity has only been addressed in EndoC-βH1 and -βH2 and only in single cell analysis in monolayers (7, 10), which are not fully informative of their properties in 3D conformation. Thus, important parameters of function remain largely unknown. Extracellular electrophysiology such as micro-electrode arrays (MEA) offers a direct and non-biased approach on whole islet characteristics. Recorded slow Potentials (SP) (11, 12) are multicellular events, representing summations of synchronized plateau depolarizations, and strictly depend on gap junction coupling by Cx36 in islet cells. Their activity is mechanistically linked to islet β-cell secretory activity and their amplitude reflects the degree of coupling (10, 11, 13–15). This allows an unbiased evaluation at a millisecond resolution without modelling and does not require genetic or chemical modifications. Furthermore, the electrical activity determined by MEAs is closely correlated to secretion (15), and its glucose concentration dependency enables small glucose-dependent increases to be distinguished in both human and murine islets (11, 14–16).

Native islets are organoids and considerable effort has been applied to assemble dispersed clonal β-cells in 3D aggregates or spheroids. Such an assembly should increase contacts between β-cells and electrical coupling between β-cells mediated by connexin 36 (Cx36) is of importance for physiological responses (17, 18). Cx36 form gap junctional channels that provide electrical coupling between β-cells and influence synchronization (19–21). Cx36 mediated coupling not only entrains cells upon arrival of a stimulus, but also dampens hyperactive cells and thus reduce spontaneous activity (22–24) which results in an improved signal/noise ratio. In contrast to primary β-cells, connexin 36 expression is generally low in β-cell lines (25).

A number of approaches have been used to generate spheroids from clonal β-cells (26, 27) such as specific media and plating in proprietary wells or microgravity (hanging drop) for human EndoC-βH cells (28–30) or rodent β-cell lines (31–34). Since recording spheroids by MEAs requires electrical contact, this precludes certain methods of spheroid formation such as coculture with endothelial cells (35) or encapsulation (36). However, using electrical activity as read-out for activity and coupling offers certain advantages as compared to fluorescent approaches such as absence of bleaching, use of chemical or genetic probes, bias by complex analysis algorithms or destructive analytical methods. It is also easier to miniaturize and to multiplex, and signals can even be analyzed online (14, 37).

Using 3D spheroids and MEAs we have now determined effective coupling in EndoC-βH1 and EndoC-βH5 cells. Our data indicate that stimulus-dependent coupling is considerably enhanced in 3D spheroids thus providing a base for their improved activity. In contrast, a widely used rat clonal β-cell line, the INS-1 derivative 832/13, did not form stable spheroids. Nevertheless, responsiveness and activity were considerably promoted by Cx36 overexpression. Therefore, both models may provide paradigms to explore mechanisms and test drugs that rely on physiological β-cell coupling.

## 2 Materials and methods

### 2.1 Materials

EndoC-βH1 cells (5) were kindly provided by Human Cell Design (Toulouse, France), EndoC-βH5 cells were purchased from Human Cell Design (Toulouse, France) and cultured according the manufacturer’s instructions. INS832/13 cells (38) were cultured as described previously (39, 40). IBMX, forskolin and glibenclamide were purchased from Sigma, GLP-1 from Bachem (Bubendorf, Switzerland). The following primary antibodies were used (all at 1:100 dilution in immunofluorescence, 1:1000 in immunoblots): CX36 mouse anti-human (Invitrogen, clone 1E5H5), rabbit recombinant ANTI-FLAG M2 antibody (Invitrogen 710662), guinea pig anti-bovine insulin (Linco, St. Charles, MO, USA), monoclonal anti-insulin (Sigma, clone K36AC10), monoclonal anti-glucagon (Sigma, clone K79bB10), polyclonal goat anti-somatostatin (Santa Cruz, sc-7819), monoclonal anti-GFP, monoclonal anti-SNAP-25 (SP12, Sternberger Monoclonals) or monoclonal anti-VAMP2 (Synaptic Systems, Göttingen, Germany). The following secondary antibodies were used: anti-mouse or anti-rabbit HRP (dilution1/2000; GE Healthcare); anti-mouse or anti-rabbit alexa568 (dilution 1/300; Invitrogen A11012 and A11031), anti-goat TMR, donkey anti-guinea pig (Jackson Laboratories, Bar Harbor, ME, USA). Note that two other primary polyclonal antibodies did not provide any reliable signal in islets or brain for Cx36 (Invitrogen 701194 and 516300). pLenti-C-Myc-DDK (RC210158L1; carrying the ORF of human CX36; GJD2; NM_020660) was obtained from Origene (Rockville, Md, USA).

### 2.2 Cell Culture and spheroid formation

EndoC-βH1 and EndoC-βH5 cells (5) were cultured according to the manufacturers protocol in OPTIβ1 (Human Cell Design, Toulouse, France). INS-1 832/13 cells were cultured as described previously (41, 42) and primary mouse islets (male C57BL/6, age 16–24 weeks) were prepared and cultured as published (12, 15, 43). Spheroids were formed in complete medium either by hanging drop for 5 days in 30 µl containing 500 islet cells or using a commercial plate (Sphericalplate 5D, Kugelmeiers; Erlenbach, Switzerland) with indicated numbers of clonal cells. Physical stability was tested by 10 times pipetting trough 200 µl tips and visual inspection with a microscope. Spheroids were considered as stable if no disaggregation was observed. Spheroid dimensions were determined on microscopic images using ImageJ v1.53. 2D experiments were conducted in largely confluent monolayers.

### 2.3 Viral transduction and quantitative PCR

Lentiviral vector production was done by Vect’UB of the Bordeaux University. Lentiviral vector was produced by transient transfection of 293T cells according to standard protocols. In brief, subconfluent 293T cells were cotransfected with lentiviral genome (psPAX2) (44), with an envelope coding plasmid (pMD2G-VSVG) and with vector constructs. Viral titers of pLV lentivectors were determined by transducing 293T cells with serial dilutions of viral supernatant and EGFP expression was quantified 5 days later by flow cytometry analysis. For transduction of INS-1 cells, 750.000 cells were incubated in 500 µl of RPMI and 5 MOI of corresponding viral particles overnight, washed and placed in complete RPMI medium for 5 days prior to plating. INS 832/13 had been transduced at population doubling number (PD) 60, cultured for 10 more PDs and maintained until PD 100.

### 2.4 Secretion assays and immunocytochemistry

Static secretion assays were performed as described (45) using Krebs-Ringer bicarbonate HEPES buffer (KRBH, concentrations in mM, 135 NaCl, 3.6 KCl, 5 NaHCO_3_, 0.5 NaH_2_PO_4_, 0.5 MgCl_2_, 1.5 CaCl_2_, 10 HEPES, 0.1% w/v BSA, pH 7.4) and hormone release was determined using commercial ELISAs (Insulin or glucagon; Mercodia, Uppsala, Sweden) as previously (40, 46). Secretion data were expressed as percent of total hormone content (obtained by acid/ethanol extraction at the end of the experiment) and calculations considered the amount secreted during the experiment. Immunocytochemistry was performed as described (41) and images acquired with a CAMSCOP CMOS camera (SCOP-Pro, Ballancourt, France) linked to an inverted fluorescent microscope (TE 200, Nikon; Champigny, France).

### 2.5 Electrophysiology

MEA recordings (60Pedot-MEA200/30iR-Au-gr, Ø30 µm, 200 µm inter-electrode distance; MCS, Tübingen, Germany) were performed at 37°C in solutions containing (in mM) NaCl 135, KCl 4.8, MgCl_2_ 1.2, CaCl_2_ 1.2 (or 2.5 in the case of INS cells), HEPES 10 and glucose as indicated (pH 7.4 adjusted with NaOH) (11, 15, 16, 39, 47). MEAs were coated with Matrigel (2% v/v) (BD Biosciences, San Diego, CA) prior to seeding of cells, spheroids or islets. Electrodes with noise levels >30 µV peak-to-peak were regarded as artefacts, connected to the ground and not analyzed. Extracellular field potentials were acquired at 10 kHz, amplified (gain 1100–1200) and band-pass filtered at 0.1–3000 Hz with a USB-MEA60-Inv-System-E amplifier (MCS; gain: 1200) or a MEA1060-Inv-BC-Standard amplifier (MCS; gain: 1100) both controlled by MC_Rack software (v4.6.2, MCS) (12, 15). Analysis of the signals was carried out with the MC_Rack software (v4.6.2, MCS). Signals were filtered with a low pass at 2 Hz in order to isolate the SPs and with a bandwidth of 3 to 700 Hz to isolate the AP. The peak detection module by thresholding of the software was used, with thresholds set by default at -1.8 µV for SP and -13 µV for AP. The minimum time between 2 events was set at 300 ms for SP and 10 ms for AP.

### 2.6 Quantitative real-time PCR

Quantitative PCR was performed as described previously (40). *YHWAZ* (Tyrosine 3-Monooxygenase/Tryptophan 5-Monooxygenase Activation Protein Zeta) and *GAPDH* were used as reference genes. *FAP* (Fibroblast activation protein, alpha), *IRX2* (iroquois homeobox 2) and *GCG* (glucagon) were used as marker genes for β-cells (48). Details and primers used are given in **Supplementary Table 1**.

### 2.7 Statistics

Data are presented as means and SD except for mean traces where SEMs were given to enhance readability. Gaussian distributions were tested by Shapiro-Wilk test and one-way ANOVA with Tukey *post hoc* or nonparametric Dunn tests were used; n corresponds to the number of electrodes recorded (from 3 distinct experiments).

## 3 Results

### 3.1 Human EndoC-βH1 cell spheroids

**Figure 1:**
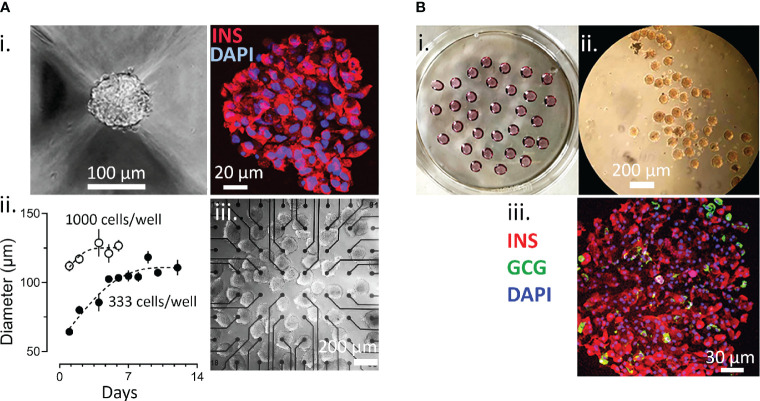
Generation of Spheroids. **(A)** Generation of EndoC-βH1 3D spheroids. i, image of spheroid formed and staining for insulin (red) and with DAPI (blue); ii, time course of spheroid formation and size at different cell numbers; iii, EndoC-βH1 3D spheroids on a micro-electrode array. **(B)** formation of islet spheroids. i petri with hanging drops; ii, 3D islets formed; iii, staining for insulin, glucagon and with DAPI.

Spheroids of human EndoC-βH1 cells were generated using micro-structured plastic wells for culture and the assembly and growth properties were tested first. As given in **Figure 1A**.i, EndoC-βH1 cells formed round and regular spheroids with a rather homogenous staining for insulin. Different spheroid sizes were monitored according to initial cell number seeding and to incubation time. Based on our experience with the culture of islets, which are native spheroids, we opted for an intermediate diameter of 103 + 8 µm obtained after 7 days (**Figure 1A**.ii) and used the corresponding protocol for all further experiments. These spheroids were mechanical stable after repetitive pipetting and conserved their spheroid form during culture on microelectrode arrays (**Figure 1A**.iii). Pseudoislets from dispersed primary mouse islet cells were prepared by the hanging drop method as this approach uses smaller quantities of cells (**Figure 1B**).

**Figure 2:**
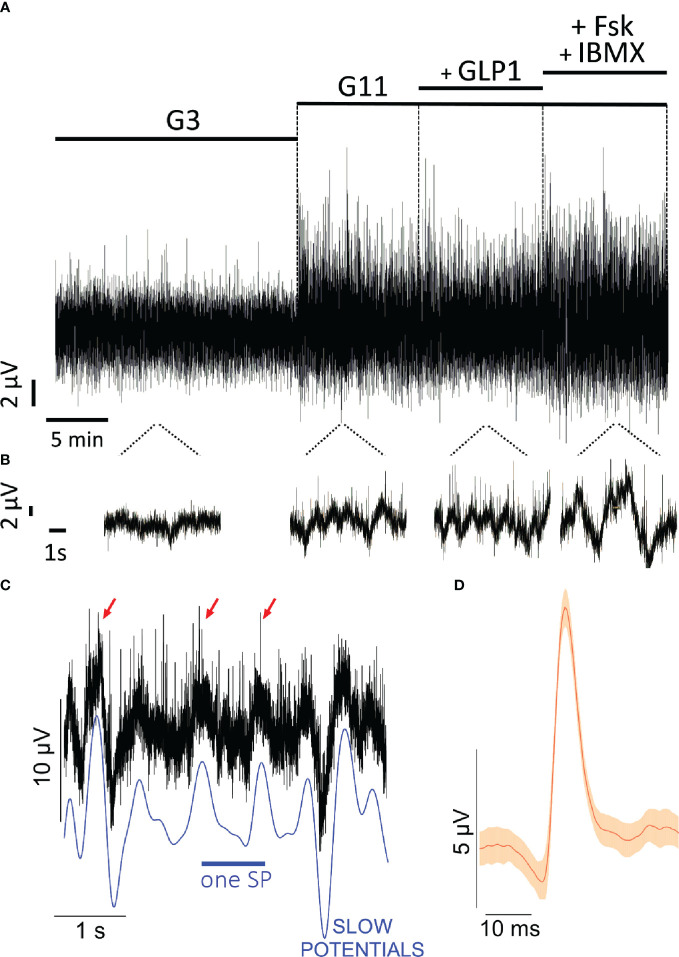
Extracellular electrophysiology of EndoC-βH cells on microelectrode arrays. **(A)** Original recordings at 3m M glucose (G3), 11 mM glucose (G11), 11 mM glucose with 50 pM GLP-1 (+ GLP1) or with 1 µM forskolin and 0.1 mM IBMX (+FSK + IBMX). Given is the example from one electrode. **(B)** snippets of recording in B on extended timescale. **(C)** example of slow potentials (SP) and action potentials (AP) from recordings shown in **(A)**. The blue line given below the recordings indicates the slow potentials, some action potentials are marked by red arrows. **(D)** mean form of action potentials (red) and 99% confidence intervals (orange) from all action potentials in the recording shown in B (n= 44; mean amplitude 8.7 µV, mean duration 35 ms).

Monolayers and spheroids were investigated using micro-electrode arrays to capture changes in extracellular field potentials induced by ion fluxes. Recordings at kHz frequency yield a large amount of data points (about 10^9^ data points for a standard experiment) and an example of raw recordings for one electrode in testing EndoC-βH1 spheroids is given in **Figure 2**. Increasing glucose concentrations from 3 to 11 mM considerable increased the amplitude in the recordings (**Figure 2A**) which seems slightly enhanced in the presence of GLP-1 and especially of forskolinforskolin/IBMX. Extension of the time scale (**Figure 2B**) allows to distinguish wave forms, the slow potentials, and superimposed very short spikes, action potentials (APs, **Figure 2C**). Slow potentials have a duration of 100 ms and more, which implies that their maximal possible frequency is around 1 Hz. The multicellular SPs recorded here represent summations of synchronized plateau depolarizations of β-cells, require connexin-expression (11, 15). Their amplitude reflects the extent of physiological important ion coupling between β-cells (15), a hallmark of physiological β-cell behavior (17, 49). Analysis of the form of APs (**Figure 2D**) reveals unitary events of 35 ms and they were shorter than APs previously observed by MEA in primary islets (12, 15).

**Figure 3:**
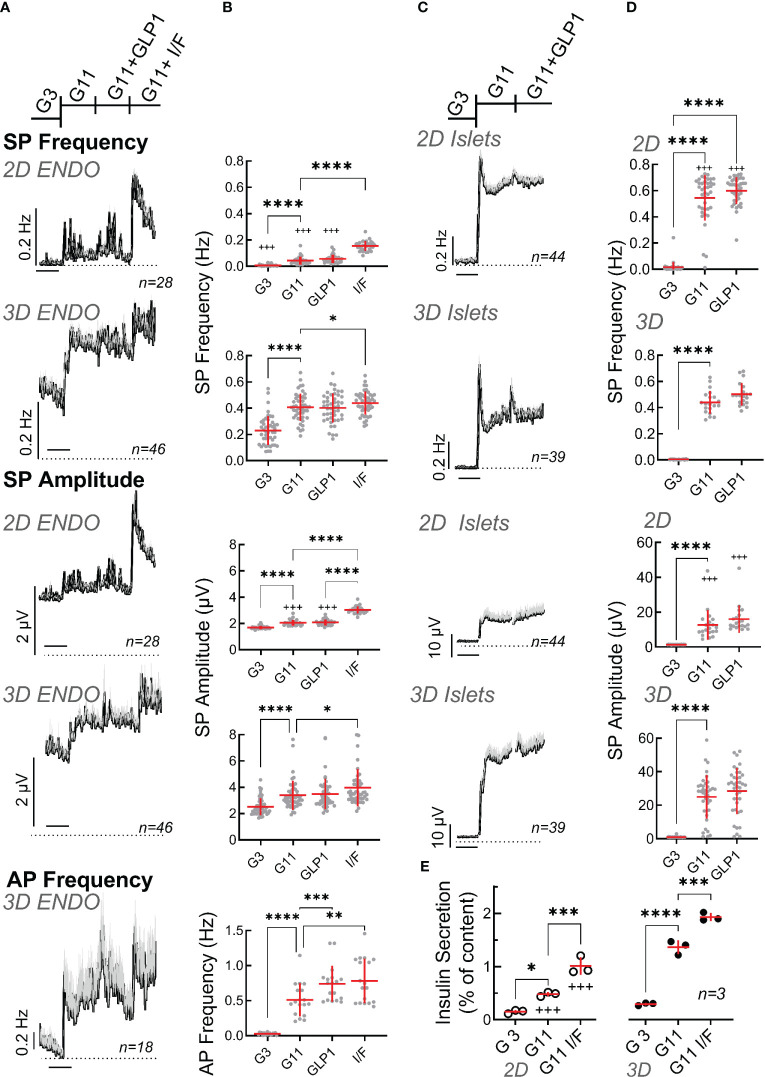
Functional characterization of spheroids from EndoC-βH1 cells or primary mouse islets. **(A)** Recording of monolayer (2D) or spheroids (3D) of EndoC-βH1 cells seeded on micro-electrode arrays and exposed to Glucose (3 mM, G3; 11 mM, G11), GLP-1 (50 pM) in the presence of 11 mM glucose (GLP1) or IBMX (100 µM) and forskolin (1 µM) in the presence of 11 mM glucose (I/F). Mean traces of slow potential (SP) frequency and amplitudes as well as action potential (AP) frequency are given; mean, black, SEM grey. Time bars equal 20 min (in all traces). **(B)** Statistical evaluations of the curves of A (mean values). **(C)** Recording of monolayer (2D) or reassembled spheroids (3D) of primary mouse seeded on micro-electrode arrays. Abbreviations for conditions and statistical tests as in **(A)**. **(D)** Statistical evaluations of curves of C (mean values). **(E)** Insulin secretion (static incubations) of monolayer (2D) or spheroids (3D) of EndoC-βH1 cells during 1h incubation, abbreviations as in **(A)** ANOVA and Tukey posthoc test; *, 2p<0.05; ** 2p<0.01, *** 2p<0.001, ****2p<0.0001; 2D vs 3D, ^+++^ 2p<0.001. n, given in corresponding panels.

We next examined electrical activity of EndoC-βH1 cells by comparing 2D monolayer versus spheroids on microelectrode arrays. Raising glucose from 3 to 11 mM significantly increased SP frequency and amplitude in 2D culture and in spheroids and the mean effect was significantly more pronounced in the latter (**Figures 3A, B**). Interestingly EndoC-βH1 spheroids had a higher basal activity (3 mM glucose) and thus fold increase between G3 and G11 in terms of mean frequency was more pronounced in monolayers (5.7 fold vs 1.8 fold in spheroids), but stronger in terms of amplitude in spheroids (1.6 in spheroids vs 1.2 fold in monolayers). The further addition of the incretin glucagon-like peptide 1 (GLP-1) at the physiological concentration of 50 pM induced a slight further increase in both cases which did, however, not reach significance. Increasing cellular cAMP levels by the direct adenylate cyclase activator forskolin and the phosphodiesterase inhibitor IBMX, in the presence of 11 mM glucose, significantly increased frequencies as compared to 11 mM glucose alone in 2D culture and spheroids. Similar effects of forskolin/IBMX in the presence of glucose were observed for amplitudes, and the relative effects were more pronounced in 2D cultures, as they were less responsive to glucose alone, but the absolute effect was stronger in spheroids (**Figures 3A, B**).

In contrast to the very robust SP signals, single cell action potentials are more difficult to detect in conventional MEAs and it is very difficult to reliably determine their amplitudes cannot (47). Moreover, as only the frequency but not the amplitude of APs varies with glucose stimulation (12), we only analyzed their frequency. In 2D cultures we were not able to identify APs with certainty. In contrast, recordings of spheroids clearly showed APs which increased with a raise in glucose concentration and significant effects of GLP-1 as well as effects of forskolin/IBMX were observed as compared to elevated glucose alone.

As a comparison to EndoC-βH1 cells we examined also mouse islet cells, either as dispersed single cell 2D culture or after reaggregation in spheroid pseudoislets (**Figures 3C, D**). In both cases, large effects were present in terms of frequency and amplitude when increasing glucose from 3 to 11 mM and the glucose-induced increase in frequency was clearly biphasic described previously (15). A small transient effect was observed for GLP-1 (50 pM) in the presence of 11 mM glucose. As already observed for the EndoC-βH1 cell line, the effect of glucose on primary islet cells was more pronounced in terms of frequency in monolayers (139 vs. 89 fold) and stronger in terms of amplitudes in spheroids (25 vs. 8.9 fold).

Next, we measured insulin secretion from 2D cultures and 3D EndoC-βH1 spheroids (**Figure 3E**). Glucose-induced stimulation was clearly apparent as well as further potentiation by IBMX/forskolin. Similar to electrical activity, basal release and stimulated insulin secretion was more pronounced in spheroids as compared to 2D cultures. Thus, the stimulation index increased from 2.6 in monolayers to 4.5 in spheroids (see **Supplementary Figure 1**). For comparison, we also measured static secretion in primary mouse islets were a rise in glucose from 0 to 3 and 11 mM increased insulin secretion from 0.05 + 0.02 to 0.07 + 0.02 and 1.15 + 0.19 (percent of content, n=5; stimulation index SI 16.4).

### 3.2 Human EndoC-βH5 cell spheroids

We subsequently tested an optimized EndoC-βH version (**Figure 2**), the EndoC-βH5 cells, known for their improved function (9). EndoC-βH5 easily formed stable spheroids (**Supplementary Figure 2**) and a mean diameter of 120 µm was used. Immunofluorescence demonstrated the expected presence of insulin as well as the SNARE proteins VAMP2 and SNAP-25. As reported previously (9), EndoC-βH5 also stained for glucagon and somatostatin in spheroids (**Supplementary Figure 2**) as did monolayers (data not shown). The presence of glucagon was also detected by qPCR (**Supplementary Figure 3**) although transcript numbers were low and other marker genes for α-cells, such as FAP and IRX were not detectable. Note that we could not detect any glucagon in static secretion assays (data not shown). Spheroid formation in EndoC-βH5 did not change the expression of glucagon or connexin 36 (GJD2) and a small decrease in the expression of preproinsulin was apparent.

**Figure 4:**
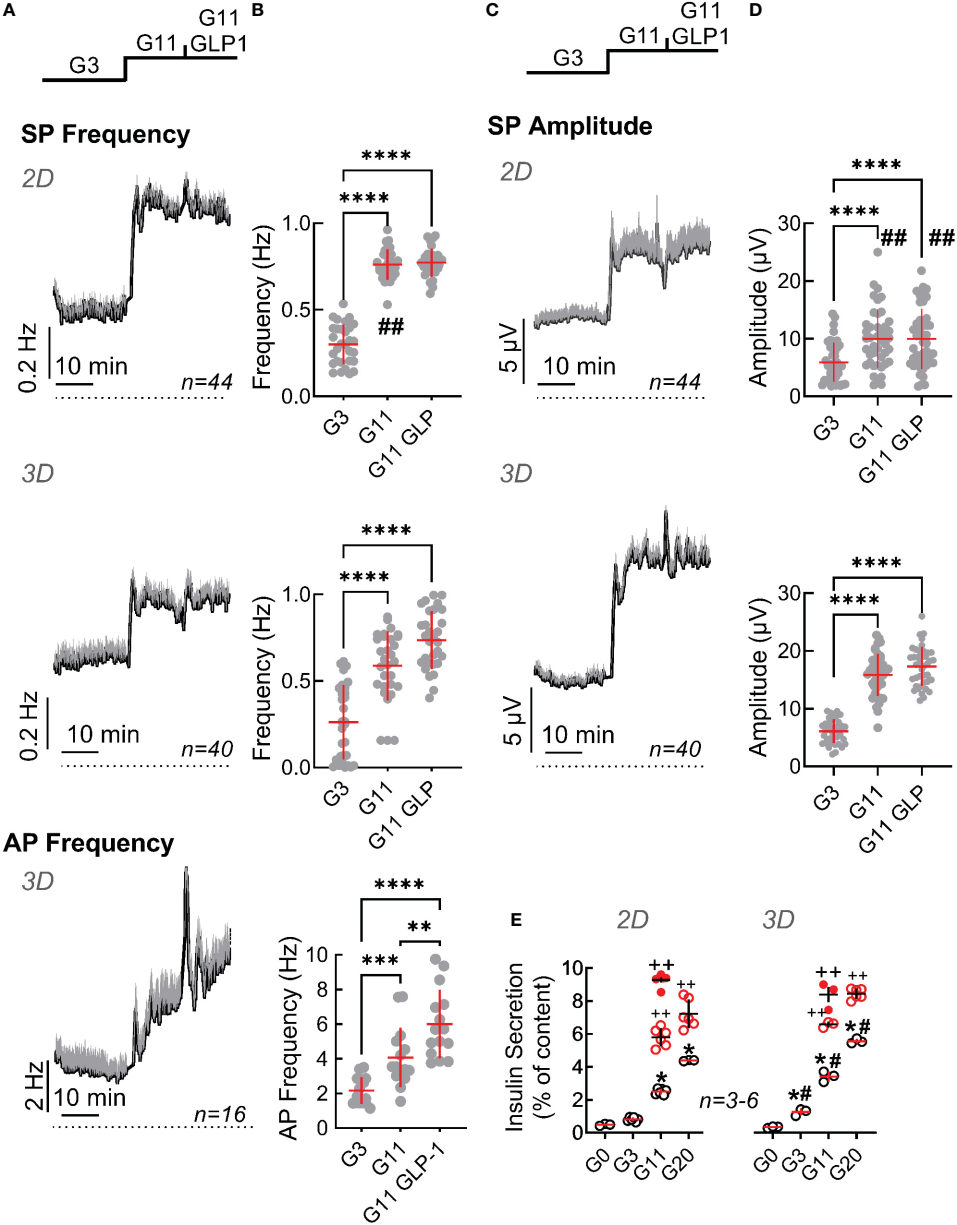
Functional characterization of monolayers and spheroids from EndoC-βH5 cells. Recording of monolayer (2D) or spheroids (3D) of EndoC-βH5 cells seeded on micro-electrode arrays and exposed to Glucose (3 mM, G3; 11 mM, G11) or GLP-1 (50 pM) in the presence of 11 mM glucose (G11 GLP1). **(A)** Mean traces of slow potential (SP) frequency as well as action potential (AP) frequency bare given; mean, black, SEM grey. Time bars equal 10 min (in all traces). **(B)** Statistical evaluations of mean frequencies of data given in **(A)**. **(C)** Mean traces of slow potential (SP) amplitudes; mean, black, SEM grey. **(D)** Statistical evaluations of data given in **(C)**. **(E)** Insulin secretion (static incubations) of monolayer (2D) or spheroids (3D) of EndoC-βH5 cells during 1h incubation, abbreviations as in **(A)** Open and filled red circles, IBMX (0.1 mM)/forskolin (1 µM) or GLP-1 (50 pM) in the presence of indicated concentrations of glucose.; ANOVA and Tukey posthoc test; **(B, D, E)** *, 2p<0.05; **, 2p <0.01; ***, 2p <0.001, ****, 2p <0.0001; Comparison 2D and 3D: #, 2p<0.05, ##, 2p<0.01; insulin secretion **(E)**, ++, 2p <0.01 as compared to the absence of GLP-1 or IBMX/forskolin; n, given in corresponding panels.

Slow potentials were more pronounced in EndoC-βH5 as compared to EndoC-βH1 in terms of frequencies and amplitudes for both, monolayers and spheroids (**Figure 4**). Similarly, action potential frequencies were higher and an effect of GLP-1 was measurable. However, an increased basal activity (at G3) was also evident in both, monolayers and spheroids, as compared to EndoC-βH1 and thus the fold increases of mean activities in terms of amplitude and frequency between G3 and G11 were comparable between the two cell lines. As observed for the EndoC-βH1 cells, spheroid formation reduced the fold increase in frequency from 3 to 11 mM glucose (2.2 fold in monolayers vs 1.8 fold in spheroids) and augmented the fold-increase in amplitudes (2.5 fold in monolayers vs 3.5 fold in spheroids). The observed temporal development of slow potentials in EndoC-βH5 cells may also suggest the presence of a first peak indicating biphasic behavior (**Figure 4**) although this was less pronounced as compared to primary mouse β-cells (see **Figure 3C**). Similar to EndoC-βH1 cells, spheroid formation enhanced insulin secretion in EndoC-βH5 (**Figure 4E**) and the cells responded well to additional GLP-1 or forskolin/IBMXforskolin. The stimulation index of insulin secretion increased from 5- (monolayers) to 10-fold (spheroids) when compared between 0 and 20 mM glucose, but between 3 and 11 mM glucose the stimulation index was around 3 fold and thus comparable to EndoC-βH1. We also noted that spheroids, in difference to monolayers, increased hormone secretion already at 3 mM glucose as compared to the absence of glucose.

### 3.3 Rat INS-1 cell spheroids

We also tried to generate spheroids from another frequently used cell line, i.e. rat insulinoma derived INS-1 832/13 cells (38, 39, 50, 51). However, these spheroids proved to be unstable to repetitive pipetting and even when handled with considerable care, spheroids rapidly disaggregated when cultured on MEAs precluding their use in 3D conformation (data not shown). Moreover, in 2D cultures a considerable number of cells or cell clusters did not respond in terms of measurable electrical activity upon increases of glucose. We therefore tested whether an increase in the expression of CX36, required for intercellular coupling and participating in adhesion (52), may improve their electrical responses.

**Figure 5:**
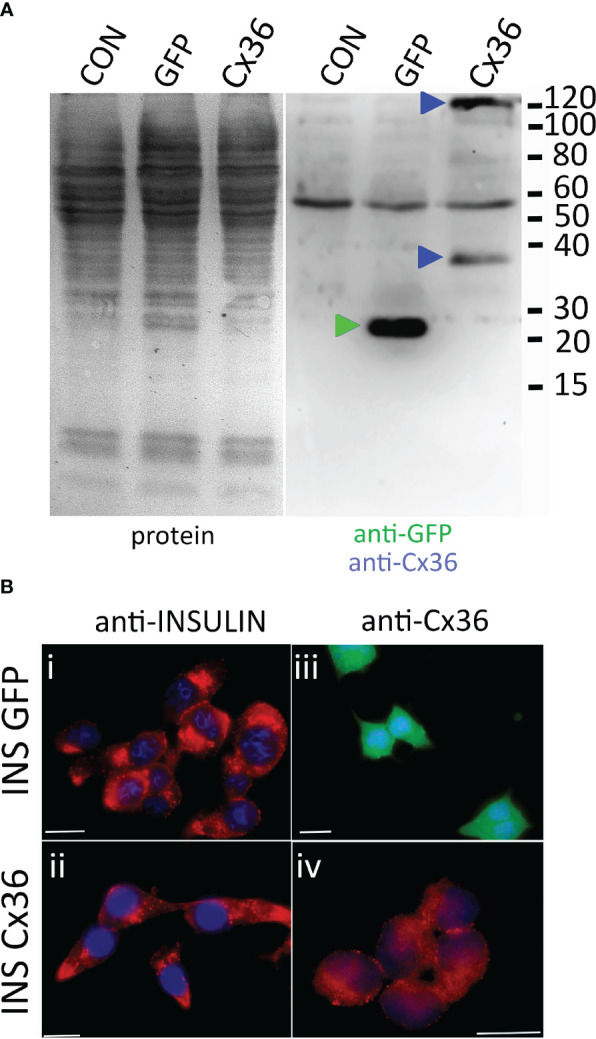
CX36 expression in transduced INS-1 cells. **(A)** immunoblot of non-transduced cells (CON) or cells transduced with either eGFP (GFP) or connexin-36 tagged with a myc-epitope (Cx 36). Left panel, protein transfer; right panel, corresponding blot co-incubated with anti-eGFP and anti-myc. Molecular weight markers are given in kDa, specifically labelled bands are indicated by correspondingly colored triangles. **(B)**, Clonal INS-1 images of INS-1 β- cells were transduced with viral particles encoding either eGFP (INS GFP, i and iii) or with Cx36 (INS Cx 36, ii and iv) and stained for insulin (i, ii) or for Cx36 (iii, iv). GFP expression was detected directly. Bars, 10 µm.

To this end INS-1 cells were transduced with viral particles encoding either GFP as a control or human connexin 36 (CX36). Immunoblot analysis of non-transduced cells and cells transduced with GFP or CX36 revealed expression of GFP or of CX36 only in the correspondingly transduced cells as bands appearing at approximately 25 kDa (GFP) or around 36 kDa as well as 110 kDa trimers (CX36) upon co-staining with GFP- and CX36 antibodies (**Figure 5A**). Human connexin was expressed intracellularly and also fine rims could be observed compatible with location at the plasma membrane (**Figure 5B**). In contrast, incubation with the anti-connexin antibody did not reveal any staining in GFP-transduced cells (**Figure 5B**).

**Figure 6:**
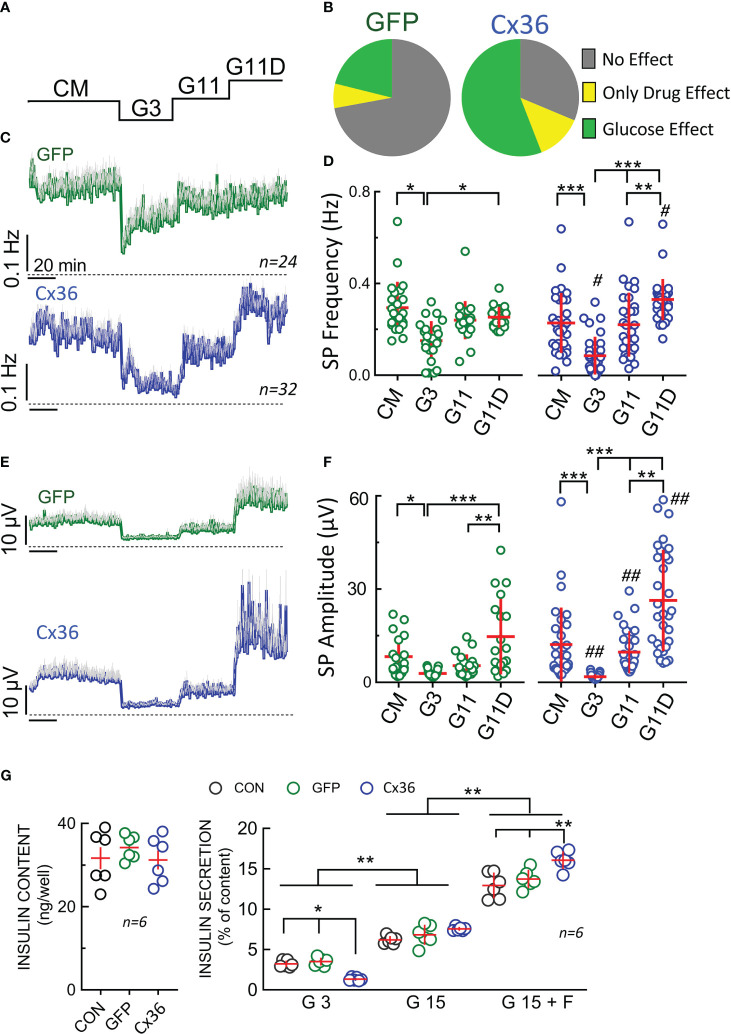
Electrophysiological analysis and insulin secretion of GFP or CX36 expressing transduced INS-1 cells. **(A)** Scheme of static incubation of INS-1 cells with culture medium (CM), 3 or 11 mM glucose (G3, G11) or 11 mM glucose in the presence of drugs (glibenclamide 200 nM, Bay K8644 1 µM, forskolin 1 µM). **(B)** relative responsivity of GFP- or Cx36 (Cx36) transduced cells expressed as absence of effect, stimulation by 11 mM glucose (versus 3 mM) or only stimulated by drugs (no effect of G11 alone; increase versus G3 by glibenclamide 200 nM, Bay K8644, forskolin 1 µM). Note that glucose-sensitive cells were always also drug sensitive. For further analysis **(C-F)** only those electrodes covered by cells were analyzed where an increase in glucose increased electrical activity. **(C)** Mean SP frequencies (+SEM) in GFP- or Cx36 transduced cells. **(D)** statistics of **(C)**. **(E)** Mean SP amplitudes (+SEM) in GFP- or Cx36 transduced cells. **(F)** statistics of **(E)**. **(G)** Insulin content and insulin secretion from non-transduced cells (CON) or GFP- (GFP) or Cx36 (Cx36) transduced cells incubated at 3 mM glucose (G3), 15 mM glucose (G15) or 15 mM glucose and 1 µM forskolin (G15 F). Statistics: Tukey or Dunn *post-hoc* tests; *, 2p<0.05; **, 2p <0.01; ***, 2p <0.001; Comparison GFP overexpression vs. CX36 overexpression: #, 2p<0.05, ##, 2p<0.01; n, given in corresponding panels.

We subsequently compared the electrical responses in terms of SP frequency and amplitude of GFP- and of Cx36-transduced cells in response to 3 or 11 mM glucose. We also used a mix consisting of the K_ATP_-channel inhibitor glibenclamide, the L-type Ca^2+^-channel agonist BayK8644 and forskolin, a direct activator of adenylate cyclases, concomitantly with glucose to obtain maximal depolarization of beta-cells (**Figure 6**). We first observed a considerable difference in their reactivity in terms of electrodes covered with cells which recorded changes in electrical activity (**Figure 6B**). Whereas in GFP-transduced cells only a minority of cells responded to an increase in glucose, more than a half were active in connexin-36 transduced cells. In fact, most of the GFP-transduced cells did not respond to glucose or glucose in the presence of stimulatory drugs (glibenclamide, Bay K8644, forskolin) in line with observations from cultures of native INS-1 cells (data not shown). We subsequently analyzed in detail the recordings from those electrodes covered with glucose-responsive cells, i.e. those cells that responded at least to an increase in glucose from 3 to 11 mM (**Figures 6C-F**). In both, GFP- or Cx36-transduced cells, the change from complete culture medium to 3 mM glucose reduced activity in terms of frequency and amplitudes. Note that complete culture medium contains 11 mM glucose and amino acids, the latter being known to enhance glucose effects (51). Interestingly, at low glucose (3 mM), SP frequency was significantly lower in Cx36-transduced cells as compared to GFP-transduced cells (**Figure 6D**). Subsequent change from 3 to 11 mM glucose increased slightly but not significantly frequency and amplitude in GFP-transduced cells whereas a significant effect was observed in CX36-transduced cells. Further exposure to stimulatory drugs significantly increased responses in CX36-transduced cells, whereas only amplitude but not frequency was enhanced in GFP-transduced cells.

Finally, we determined insulin content and secretion in non-transduced and GFP- or Cx36-transduced cells (**Figure 6G**). Under all three conditions (non-transduced, GFP-transduced or CX36 transduced cells) insulin content did not vary. Clearly, Cx36 expression reduced basal secretion (at 3 mM glucose) in Cx36 transduced cells as compared to the two other conditions. In all three cell types, an increase in glucose stimulated secretion was observed. The stimulation index (15 mM vs 3 mM glucose) amounted to 1,9 in non-transduced and GFP transduced cells, but increased to 5,7 in Cx36 transduced cells. Although Cx36-transduced cells secreted 20% more insulin than GFP-transduced cells at 15 mM glucose, the increase in GSIS was mainly due to an approximately 60% reduction in basal secretion at 3 mM glucose in Cx36-transduced cells. Forskolin in the presence of 15 mM glucose further enhanced insulin secretion and again to a greater degree extent in Cx36-transduced cells as compared to GFP-transduced or non-transduced cells.

## 4 Discussion

Our results indicate that spheroids of human EndoC-βH1 and -βH5 cells exhibit more pronounced signals in extracellular physiology than monolayers. Spheroids have higher amplitude of slow potentials on stimulation indicating a higher degree of cell-cell coupling and improving detection. This is accompanied by a considerable improvement in the glucose-stimulated insulin secretion. The lack of stable spheroid formation in rat clonal INS-1 β-cells may be compensated by the enhanced expression of connexin 36, which also improves GSIS index mainly by lowering basal electrical activity and ensuing secretion.

3D spheroids of human EndoC-βH1, -βH3 or -βH5 were generated previously using microgravity (“hanging drop”), low attachment plates or co-culture on human umbilical vein or islet-derived endothelial cells (9, 28–30, 53). These spheroids exhibited a GSIS index similar to that observed in our study. Spheroid size used here was chosen on the ground of several arguments: larger islets are known to be more prone to central necrosis (54), islets with a diameter of less than 150 μm better secrete insulin (55–57) and 100 µm is about the average islet size in humans or mice (58). Moreover, modelling suggests that islet size between 50 and 150 µm may favor network interactions that are crucial for islet function (59). The method employed here by us has the advantage of simplicity as well as controlled and reproducible spheroid size. Reproducible size is an important factor in standardization as large spheroids may undergo core necrosis (60), whereas variation in size may lead to differences in cell-cell coupling (61, 62) and insulin secretion (63). An attractive alternative, especially when using extracellular electrophysiology, may be given by cell electrophoresis of dispersed cells onto electrodes (16).

The slow potentials recorded here represent summations of synchronized plateau depolarizations of β-cells depending on connexin-36 expression and their amplitude provides an unbiased read-out of the degree of β-cell coupling (11, 15). SPs are mechanistically linked to insulin secretion (15, 40), although the precise weight of their frequency vs their amplitude has only been determined in primary islets under microfluidics and may vary in other models (15). Notably, a biomimetic algorithm, based on islet SPs, was extremely well adapted to regulate insulin delivery in a human in-silico model of type 1 diabetes, the UVA PADOVA TMDS (13, 64).

Clearly spheroid formation provided far more robust electrophysiological responses and even permitted to reliably detect action potentials which are difficult to monitor in monolayers even when using electrodes coated with a conducting polymer (16). In all EndoC-βH models and in primary islet cells we observed an increase in SP amplitude in spheroids that are most likely due to improved coupling (11, 15) and correlated with an increase in insulin secretion in the three cell models tested. The improvement in insulin secretion observed here was comparable to published data (6, 29). We do not have a ready explanation for the unexpected increase in SP frequency at 3 mM glucose in EndoC-βH1 spheroids as compared to monolayers. This may indicate that spheroids have a higher sensitivity to glucose and increased electrical activity was also mirrored by almost doubling of insulin secretion in these spheroids, which, however did not become significant. In the same vein EndoC-βH5 spheroids had a different set-point in glucose-induced insulin secretion in our hands and differing from a previous report (9). One may speculate that spheroid formation enhances the concentrations of locally released autocrine factors. It has indeed been demonstrated that paracrine secretion of ATP from primary β-cells occurs at 3mM glucose and enhances secretion (65), although we do not know whether this ATP release also occurs in EndoC-βH cells. In more general terms, our observation may pinpoint to the crucial role of δ-cells in native islets, which are absent in these spheroids, in setting the glucose threshold (66).

Notably, in EndoC-βH5 cells a biphasic pattern of electrical activity was apparent upon glucose stimulation, which is a hallmark of primary islets (15) and such a pattern had also been reported in dynamic insulin secretion assays of EndoC-βH5 cells (9). We did not observe any significant effect on slow potentials by activating the GLP-1 receptor via its native agonist at physiological concentrations. Note that we previously observed that these concentrations significantly increase slow potential amplitude and frequency in mouse and human islets although the effect was small (15). The effects of GLP-1 on action potentials observed here indicate that the hormone signaling pathway was active in the clonal EndoC-βH cells in our study. As the GLP-1 inactivating protease DDP-4 is expressed in EndoC-βH cells (67), most studies used the incretin mimetic peptide exendin-4 (6, 9, 29, 68) with one exception (69). However, we think that such a pharmacological approach yields less insight. In the same vein, glucose concentrations should be chosen carefully and we prefer a minimal glucose concentration of 3 mM to the complete absence of glucose. Although stimulation indices are more pronounced when referring to “0 glucose”, prolonged absence of glucose may considerably alter gene expression profiles (70). Similar to other clonal β-cell lines, such as INS-1 or MIN6 (71–74), EndoC-βH5 have been reported to express preproglucagon (9). We confirm the observation of a low number of transcripts (9) and we could not detect any secreted glucagon rendering unlikely its contribution to cellular activity.

We were not able to form stable spheroids using rat insulinoma INS-1 cells (38, 50), a widely used and relevant clonal β-cell model. Most spheroids published of this cell line were generally of variable sizes and poorly defined borders although the methods used for spheroid formation provided useful spheroids in another β-cell lines such as MIN6 (31, 75–77). A more recent publication reported the generation of uniform spheroids but were maintained in a device that is not compatible with electrophysiological recordings (78, 79). It is also of note that most of the previously reported INS-1 aggregates showed either a considerable right shift of glucose dependency or a stark reduction in glucose induced insulin secretion (75, 76). Cx36 overexpression increased the percentage of glucose-responsive cells and induced here a coherent pattern in electrical activity and insulin secretion largely confluent monolayers and clusters: a 2.4 fold increase in the percentage of glucose-sensitive cells, reduced basal electrical and secretory activity, a significant increase in glucose-induced SP amplitudes and insulin secretion and consequently almost doubling in glucose induced stimulation indices. These observations are in line with the general function of Cx36 in islets (19) and in INS-1 cells (80).

In conclusion, EndoC-βH1 and especially EndoC-βH5 spheroids provide a very useful model to test the effect of different variables on electrical activity and physiological cell-cell coupling by MEA analysis in line with the advocated utility in drug testing (6). Cx36-transduced INS-1 cells may be suitable, but obviously restricted to rodents. The human models may also be of interest as biological substrate for organs on chips and microorgan-based sensors for continuous nutrient sensing (37, 64, 81).

## Data availability statement

The raw data supporting the conclusions of this article will be made available by the authors, without undue reservation.

## Ethics statement

Ethical approval was not required for the study involving animals in accordance with the local legislation and institutional requirements because Use of established cell lines which have been used since a decade in research.

## Author contributions

EP: Data curation, Investigation, Software, Writing – original draft, Writing – review & editing, Formal Analysis. KL: Data curation, Formal Analysis, Investigation, Writing – review & editing, Writing – original draft. JG: Investigation, Methodology, Writing – review & editing, Writing – original draft. ML: Investigation, Writing – review & editing, Writing – original draft. PS: Writing – review & editing, Investigation, Writing – original draft. MR: Conceptualization, Formal Analysis, Supervision, Writing – review & editing, Writing – original draft. JL: Conceptualization, Data curation, Formal Analysis, Funding acquisition, Investigation, Methodology, Project administration, Resources, Supervision, Validation, Writing – original draft, Writing – review & editing.
